# Disposable Printed CamBlobs Charts for Measuring Contrast Sensitivity in Patients With Glaucoma

**DOI:** 10.1155/joph/4199453

**Published:** 2026-02-27

**Authors:** Shahina Pardhan, Rajiv Raman, Rekha Srinivasan, Suwin Hewage, John Kidd, Mapa Prabhath Piyasena

**Affiliations:** ^1^ Vision and Eye Research Institute, School of Medicine, Anglia Ruskin University, Young Street Cambridge, Cambridgeshire, CB1 2LZ, UK, anglia.ac.uk; ^2^ Department of Ophthalmology, UF College of Medicine, Jacksonville, Florida, USA; ^3^ Shri Bhagwan Mahavir Vitreo-Retinal Services, Shankara Nethralaya College Road, Chennai, Tamil Nadu, 600 006, India; ^4^ Retina Research Unit of Department of Vitreo-Retina, National Eye Hospital, Colombo, Sri Lanka

**Keywords:** agreement, CamBlobs, contrast sensitivity, diagnostic accuracy, glaucoma, Pelli–Robson

## Abstract

**Purpose:**

Contrast sensitivity (CS) is lowered in glaucoma. We aimed to assess whether a simple inexpensive CamBlobs CS test is just as effective as an established Pelli–Robson CS chart in assessing CS in people with glaucoma.

**Methods:**

This study included two‐groups of participants (27 no ocular disease and 23 glaucoma patients) who underwent CS testing using CamBlobs chart and a Pelli–Robson chart in each eye. Agreement and reliability between CamBlobs and Pelli–Robson charts were examined using Bland–Altman plots and receiver operating characteristic (ROC) curves to assess the diagnostic test accuracy. Visual field data were also obtained and compared with CS data from both charts.

**Results:**

A total of 54 eyes of control and 44 eyes of glaucoma patients were included. There were significant differences in CS between the control and glaucoma groups measured by the CamBlobs chart (*p* < 0.001) and also Pelli–Robson chart (*p* < 0.001). The area under the ROC‐curve (AUC) for CamBlobs was 0.85 (*p* < 0.001) with sensitivity at 68.2% (95% CI 53.7%–80.6%) and specificity at 85.2% (95% CI 74.2%–92.9%), and for Pelli–Robson, the AUC was 0.88 (*p* < 0.001) with sensitivity at 93.2% (95% CI 83.3%–98.3%) and specificity at 66.7% (95% CI 53.5%–78.3%) for differentiating people with glaucoma and control by measuring CS. The correlation between mean deviation of the visual fields and CamBlobs chart log CS was *r* = 0.59 (*p* < 0.001), and with Pelli–Robson chart was *r* = 0.65 (*p* < 0.001).

**Conclusions:**

CamBlobs charts are comparable to Pelli–Robson CS charts for assessing CS in people with glaucoma. CamBlobs offer a simple, portable method for CS testing beyond conventional clinical settings.

## 1. Introduction

Glaucoma is one of the leading causes of irreversible blindness worldwide [1] and is a major public health issue in a rapidly ageing population globally. Currently, 64.3 million people suffer from glaucoma worldwide, which is expected to increase to 111.8 million by 2040 [2]. A recent meta‐analysis reported that glaucoma remains one of the major causes of blindness globally, and effective strategies for early detection and treatment are therefore needed [3]. Glaucoma compromises quality of life and impairs performance of broad range of activities. As glaucoma cannot be self‐detected until a late stage, it is termed the *“silent thief of sight”.* Current clinical diagnosis of glaucoma includes patient and family history, intraocular pressure measurements, assessment of the anterior chamber of the eye, evaluation of the optic nerve head, visual field (VF) measurements and structural parameter assessment using optical coherence tomography (OCT). Considering the irreversible nature of the pathogenesis process and early asymptomatic phase, as well as undetected cases, novel approaches to examining vision functions and early detection would be beneficial. However, the process of investigating the vision functions and disease diagnosis is complex and costly and cumbersome to apply at population level using conventional methods such as perimetry and structural assessments including OCT [4]. So, a need for easier and less cumbersome measurements to test vision functions for glaucoma exists.

### 1.1. Contrast Sensitivity (CS) and Glaucoma

CS has also been shown to decrease in people with glaucoma. Our day‐to‐day activities involve viewing various objects, shadows of different surfaces and patterns, and the visual system is often required to discriminate different contrasts and adapt to ambient conditions. Under such situations, the contrast keeps changing globally, and due to the local variations across the environment [5]. People with glaucoma have difficulties in day‐to‐day activities such as light and dark adaptation, outdoor mobility, driving tasks and difficulties in reading [6–8].

Studies have reported that the ability to discriminate and adapt to contrast is affected in glaucoma [8], and CS is an important visual function to assess the quality of life of people with glaucoma [9]. Moreover, contrast changes seem to occur before significant visual damage [10], as studies have shown that CS precedes nerve fibre layer damages reflected by perimetry [6–8, 11], and hence may be a better parameter to capture functioning of the visual system, especially when the central visual acuity is within the normal limits [12]. It has been postulated that CS may have the potential for differentiating those who are at risk of developing glaucoma [11]. Most of the previous studies reported that CS decrease in glaucoma is not just due to ageing process [13], and significant differences in CS exist between glaucoma patients and control older subjects [14, 15].

Various tests have been used to examine changes in CS in glaucoma. Verbaken and Johnston have reported that 70% of their control participants had test–retest variability within ± 1 dB of the CS score (mean 38.9 dB; SD ± 3.1 dB) tested on 499 eyes using the Melbourne Edge test [16]. A study conducted by Bambo et al. using the Pelli–Robson chart test showed that CS decreases in patients with glaucoma and is significantly worse in those who have moderate glaucoma compared to those with early stages [17]. Another study including 28 patients with open‐angle glaucoma showed a moderate to profound deficit in CS among patients with open‐angle glaucoma using Pelli–Robson (median Log CS 1.35) charts and Freiburg Visual Acuity and Contrast Test (FrACT) charts (median Log CS 1.39). Wilensky et al. (2001) reported that CS measured using Pelli–Robson charts correlated with increased VF loss (*r* = 0.638 for open angle glaucoma) [18]. CS testing therefore provides valuable clinical information on functional visual performance in glaucomatous and nonglaucomatous eyes, complementing the standard screening and diagnostic methods for glaucoma [16, 19].

CamBlobs chart offer a simplified alternative to conventional CS tests that do not require administering by specialised healthcare personnel. When printed, they require minimal technical resources. CamBlobs chart uses spots and employs four alternative forced‐choice techniques which can be used on illiterate patients with glaucoma. These charts require no maintenance on repeated usage and easy‐to‐use. Spot design of the CamBlobs chart provides the ability to test the CS in a wide luminance range [20] and eliminates the possibility of letter pattern memorisation by the users. A study conducted to compare CamBlobs CS test to the near Pelli–Robson in normally sighted young adults (mean age 28 ± 4 years) reported CamBlobs CS intravisit repeatability consistent with the Pelli–Robson test (±0.20 Log CS) [20].

The current study compares data from one such newly developed CamBlobs chart (SpotChecks) with an established Pelli–Robson chart. There is a possibility of self‐administering CamBlobs CS testing at nonclinical settings and can be deployed as a tool to provide clinically relevant information on functional visual performance in glaucomatous and nonglaucomatous eyes, complementing standard screening and diagnostic tests for glaucoma, especially in resource‐limited settings. The primary aim of this study was to evaluate the performance of CamBlobs charts relative to the established near Pelli–Robson chart for the measurement of CS in people with glaucoma.

## 2. Subjects and Methods

### 2.1. Study Population

Two groups of participants were recruited; 27 normal participants with no ocular disease and 23 patients who were diagnosed with open‐angle glaucoma with intraocular pressure of ≤ 32 mm Hg in either eye and a confirmed diagnosis based on increased vertical cup–disc ratio (VCDR) or cup–disc ratio (CDR) asymmetry of ≥ 0.2, focal or diffuse neuroretinal rim (NRR) thinning, localised notching, disc haemorrhages, retinal nerve fibre layer (RNFL) defect with corresponding VF changes on Swedish Interactive Thresholding Algorithm (SITA) standard HVF 24‐2 protocol. Severity was classified using mean deviation (MD) and Visual Field Index (VFI) as follows: Mild MD > ‐6 dB, VFI > 90%; moderate MD between −6 and −12 dB; VFI 60%–90% and severe MD < −12 dB, VFI < 60%.

### 2.2. Ethics Approval

The study adhered the tenets of the Declaration of the Helsinki. The study protocol and the experimental details of the study were reviewed and approved by the Institutional Review Board and the Ethics Committee, Vision Research Foundation, Sankara Nethralaya, Chennai. Written informed consent was obtained from all study participants.

Participants were excluded if they had strabismus (Tropia), amblyopia, nerve palsy, nystagmus, pupillary abnormalities, corneal abnormalities, cataract status > NS2 and/or P1 and/or C2 (LOCS II) [21], history of ocular trauma, history of corneal or retinal surgeries, presence of posterior capsular opacification (PCO), best corrected visual acuity (BCVA) ≤ 6/12, N6 in both the eyes, presence of any macular or retinal pathologies and any evidence of visual pathway lesions.

All the participants underwent a comprehensive eye examination by an ophthalmologist, following which the CS measurements using CamBlobs chart (specifications given below), and Pelli–Robson CS chart (Precision Vision, USA) were obtained. CamBlobs and Pelli–Robson charts used in the current study are designed for use at near.

#### 2.2.1. CS Measurement Protocol and Data Collection

All the measurements were carried out in a standard room illumination maintained over 200 lux, and participants were instructed to wear their near vision correction with testing carried out at their habitual working distance. CS was tested with CamBlobs chart and Pelli–Robson CS chart in each eye. With normal participants, the right eye was tested first, and for participants with glaucoma, the better eye was first tested.

#### 2.2.2. CamBlobs Chart

**FIGURE 1:**
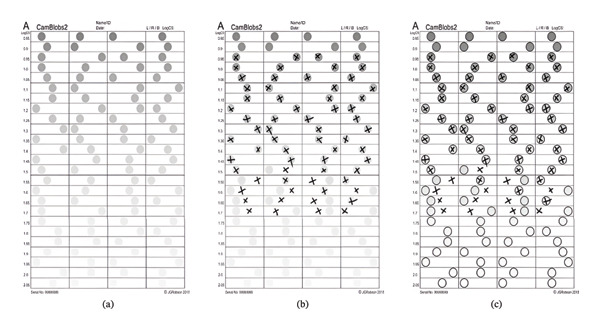
CamBlobs experimental illiterate contrast sensitivity chart ((a) an unmarked chart, (b) a chart marked by a subject and (c) the marked chart with a transparent overlay showing the location of the of the printed spots).

The charts were inkjet‐printed using black and neutral grey pigment inks on A4 sheets (210 × 297 mm) of heavy‐weight (200 gsm) matte white paper that had no added optical brightening agents (OBAs) [22]. Charts were nearly covered by a grid of fine black lines that outline an array of horizontally elongated rectangles arranged in four columns and 25 rows. CamBlobs chart consists of 25 rows of four rectangles with grey spots (9 mm in diameter) randomly positioned along the row printed on A4‐sized charts. The contrast of each row is the same and decreases gradually in 0.05 log units (0.5 dB) than the previous row above it. The log Weber contrast (log CS) ranges from −0.85 log dB to −2.05 log dB (Weber contrast as a percentage from 14.1% at the top line to less than 1% at the bottom line of the chart) (see Figure 1).

The CamBlobs charts were clipped to a board that was held by the subject at a nominal distance of 40 cm, but subjects were free to hold it at any distance not too far from this at which it was seen most clearly, and it was convenient for them to mark the chart with a pen. The participants were instructed to view the charts monocularly at their habitual working distance and with a reading aid (if required), and to indicate with a cross ‘X’ the rectangle they perceived to contain the grey spot. Participants marked the position of all four spots in each row that they could see. If in doubt, they were instructed to mark the position of spots they guessed were the correct ones, as instructed by the manufacturer. The last row on which there were less than two errors (i.e., on which more than two spots were correctly located) was assumed to indicate the subject’s contrast threshold. The inverse of the contrast of the spots in this row was recorded as the subject’s CS, which the logarithm of this value (logCS = −logC) was printed on the charts.

#### 2.2.3. Pelli–Robson CS Chart

Near Pelli–Robson CS chart consists of eight lines of standard optotypes of the same letter size. Every line consists of two groups where each group consists of three letters of the same contrast, and the contrast of every group decreases successively by a factor of 0.15. The CS ranges from 0.00 log dB to 2.25 log dB. The Pelli–Robson charts were viewed at a distance of 40 cm, and the test was carried out by the examiner scoring them according to the standard instruction. The participants were instructed to view the charts monocularly at their habitual working distance and reading aid (if required) and read aloud each group from the top line until they saw the faintest group. The lowest contrast level group, in which participants correctly identified at least two optotypes, was recorded as the thershold. Similar protocol was followed for the other eye measurement.

#### 2.2.4. Statistical Analysis

All the details of the participants and relevant information were entered in a predesigned Microsoft Office Excel 2016 (Microsoft Corp, Redmond, WA) sheet. Normality tests for all the continuous variables and appropriate parametric/nonparametric tests were performed.

Agreements between CamBlobs charts and Pelli–Robson charts were estimated using Bland–Altman plots for the control sample and participants with glaucoma. The average agreement was estimated using the mean difference of CS estimates using CamBlobs and Pelli–Robson charts, and agreement for each eye was summarised using the limits of agreement (LoA). As measures of the two methods, i.e., CamBlobs and Pelli–Robson, were expected to agree absolutely, for the intraclass correlation (ICC) calculation, the two‐way mixed effects absolute agreement method was used. Type 1 error was kept at 5% level, and all the tests used were two‐tailed. Performance of measuring CS was explored using the discriminative ability of identifying glaucomatous with nonglaucomatous eyes among study participants as a proxy measure, using Pelli–Robson and CamBlobs charts. Statistically, this were assessed using receiver operating characteristic (ROC) curves, with VF parameters (i.e., MD 24‐2 including 10‐2 in advanced cases and VFI) as the gold standard of identifying glaucomatous eyes. Paired design ROC curves with CS from each of the test (Pelli–Robson and CamBlobs) were generated. From these, the optimal cutoff for log CS (*Yoden index,* i.e., maximum potential effectiveness of CS for differentiating glaucomatous and nonglaucomatous eye) and logCS point with minimal misclassification rate (*Index of Union*, i.e., the point where there is minimal difference between the sensitivity and specificity) were determined for each of the two tests and then applied in a second model to assess the discriminative ability of identifying glaucomatous and nonglaucomatous eyes using CamBlobs chart when compared to the Pelli–Robson (Pelli–Robson as the reference standard in the second model).

## 3. Results

### 3.1. Demographic Details

A total of 54 eyes of control participants (*n* = 27) and 44 eyes of glaucoma patients (*n* = 23) were considered for analysis. The mean age (SD) of control participants was 47.6 (8.68) years, and that of the glaucoma group patients was 52.6 (15.7) years which was not significantly different (*p* = 0.186; independent *t*‐test, level of significance *p* < 0.05). The glaucoma group participants were classified into mild, moderate and severe VF defects, based on the Hodapp–Anderson–Parrish (HAP) criteria [23]. Of the 44 eyes in the glaucoma group, 52.3% of eyes (*n* = 23) had mild VF defect, 9.1% of eyes (*n* = 4) had moderate visual defect and 38.6% eyes (*n* = 17) had a severe visual defect.

Table 1 shows the clinical variables of the two groups. The average (best corrected LogMAR acuity for the control group was 0.00 (SD 0.00), which was statistically different to the glaucoma group 0.06 (SD 0.15).

**TABLE 1:** Descriptive details of the clinical variables.

Variables	Study characteristics	Normal (*N* = 54)	Glaucoma (*n* = 44)	*p* value^∗^
Best corrected distance visual acuity (mean ± SD	LogMAR visual acuity	0.00	0.06 ± 0.15	0.001
Visual field indices (24‐2) (Mean ± SD)	Foveal threshold Visual field Index Mean deviation	NA	32.53 ± 3.19 68.83 ± 32.15 −11.79 ± 9.75	NA
Near contrast sensitivity (mean ± SD)	CamBlobs chart (log dB) Near Pelli–Robson contrast sensitivity chart (log dB)	1.66 ± 0.07 1.76 ± 0.09	1.44 ± 0.22 1.51 ± 0.26	< 0.001 < 0.001
Near contrast sensitivity (mean ± SD)	Comparison of mean values of near contrast sensitivity (CamBlobs vs. Pelli–Robson) (log dB)^†^	SE 0.016 *p* < 0.0001	SE 0.051 *p* = 0.1763	NA
Correlation (*r*) between contrast measures and mean deviation of visual field test^‡^	CamBlobs chart Near Pelli–Robson contrast sensitivity chart	NA	0.59 0.65	< 0.001 < 0.001

Abbreviations: MAR = minimum angle of resolution, NA = not applicable, and SD = standard deviation.

^∗^Independent *t* test, significant at *p* < 0.05.

^†^Paired *t* test, significant at *p* < 0.05.

^‡^Intraclass correlation.

CS was significantly different between the glaucoma and normal participants with both the CamBlobs (*p* < 0.001) and near Pelli–Robson CS charts (*p* < 0.001). Among the normal participants’ mean near CS values of CamBlobs versus Pelli–Robson was significantly different (*p* < 0.0001), while it was not significantly different among participants with glaucoma (*p* = 0.1763).

A significant correlation was obtained between the MD from the VF and CS using CamBlobs chart log CS (*r* = 0.59, *p* < 0.001) and with Pelli–Robson chart (*r* = 0.65, *p* < 0.001), suggesting that CS testing is a potential functional measure of differentiating glaucomatous and nonglaucomatous eyes; ascertaining CamBlobs charts have similar performance compared to established Pelli–Robson on measuring CS among people with glaucoma.

Agreement and reliability between the CamBlobs and near Pelli–Robson CS charts were estimated using the Bland–Altman plots and ICC, as shown in Table 2. There was a good agreement between CamBlobs and Pelli–Robson charts CS data among the people with glaucoma (ICC 0.89; 95% CI 0.77–0.94). The Bland–Altman graph with subcategorisation based on control and glaucoma groups is given in the Supporting File 1.

**TABLE 2:** Agreement and reliability between the CamBlobs and near Pelli–Robson contrast sensitivity charts.

Study groups	MD	LOA	ICC	95% CI
Overall (*N* = 98)	−0.09	−0.30 to 0.13	0.88	0.60 to 0.95
Control group (*N* = 54)	−0.1	−0.24 to 0.04	0.51	−0.23 to 0.80
Glaucoma group (*N* = 44)	−0.06	−0.34 to 0.22	0.89	0.77 to 0.94

*Note:* ICC: intraclass correlation (absolute agreement; two‐way mixed‐effects model).

Abbreviations: CI = confidence interval, LOA = limits of agreement, and MD = mean difference.

The mean difference between the CS values between the two charts was −0.09 log dB, and the ICC was 0.88 (95% CI 0.60–0.95) for the whole group, meaning a strong agreement between CamBlobs and Pelli–Robson for measuring CS. The mean difference between the two charts were −0.1 log dB for the control group (ICC: 0.51 [95% CI 0.23–0.80]) and −0.06 log dB for the glaucoma group (ICC: 0.89 [95% CI 0.77–0.94]).

### 3.2. ROC Curves

#### 3.2.1. Comparison of CS Testing Findings of CamBlobs and Near Pelli–Robson vs. VF Findings

**FIGURE 2:**
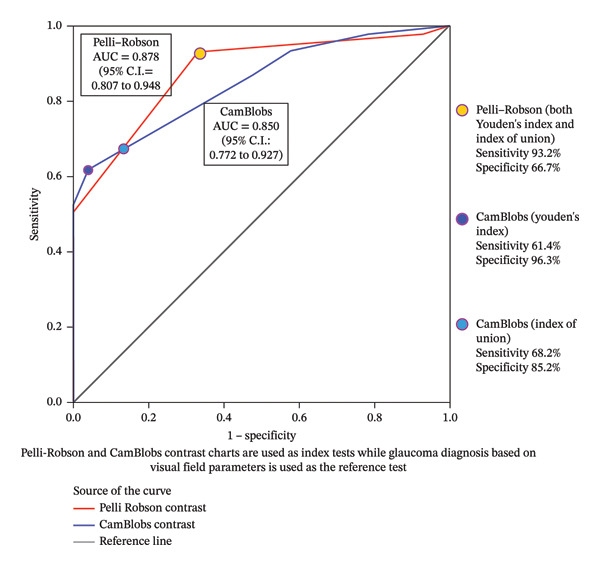
Receiver operating characteristics curve for differentiating people with glaucoma and controls based on log CS measurements of CamBlobs and Pelli–Robson compared to VF findings.

The area under the curve (AUC) was plotted to explore performance of CamBlobs vs. Pelli–Robson charts through the differentiating ability between people with glaucoma and controls, using CS testing as a proxy measure. In this ROC model, VF parameters were used as the gold standard (i.e., MD 24‐2 including 10‐2 in advanced cases and VFI), and based on an assumption that VF parameters of the control sample is 100% within normal limits (Figure 2). The AUC for CamBlobs CS charts was 0.85 (SE 0.040, *p* < 0.001 and 95% CI 0.77–0.93) with the highest effective sensitivity at 61.4% (95% CI 46.6%–74.8%) and specificity at 96.3% (95% CI 89.0%–99.4%) using the Youden’s index. Sensitivity improved to 68.2% (95% CI 53.7%–80.6%) and specificity to 85.2% (95% CI 74.2%–92.9%) when the Index of Union criterion was applied. Coordinates of the ROC curve of CamBlobs using both methods are given in Supporting File 2. The AUC for Pelli–Robson CS chart was 0.88 (SE 0.036, *p* < 0.001 and 95% CI 0.81–0.95) with the highest effective sensitivity at 93.2% (95% CI 83.3%–98.3%) and specificity at 66.7% (95% CI 53.5%–78.3%), using both Youden’s index and Index of Union criteria. Coordinates for the ROC curve of Pelli–Robson using both methods are given in Supporting File 2. The substantial overlap of the 95% confidence intervals between CamBlobs and the Pelli–Robson chart indicates comparable CS testing performance between the two charts, with no evidence of a statistically significant difference.

#### 3.2.2. Comparison of CS Testing of CamBlobs vs. Near Pelli–Robson

**FIGURE 3:**
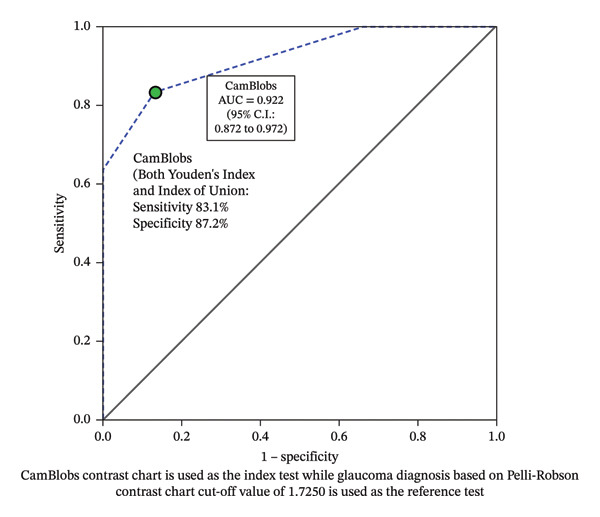
Receiver operating characteristics curve for differentiating people with glaucoma and controls based on log CS measurements of CamBlobs compared to Pelli–Robson findings.

In a different model (see Figure 3), we assessed the performance of CS testing through the diagnostic test accuracy of differentiating people with glaucoma vs. controls using log CS measurements of CamBlobs, with the Pelli–Robson charts as a reference standard (at the optimum diagnostic test accuracy cutoff 1.7250 Log CS value for the Pelli–Robson chart for differentiating people with glaucoma vs. controls). The log CS cutoff for differentiating people with glaucoma vs. controls was considered at optimum cutoff of log CS = 1.625 for the CamBlobs chart, and the AUC for the CamBlobs chart was 0.922 (SE 0.025, *p* < 0.001 and 95% CI 0.87–0.97). In this ROC curve model, the highest effective sensitivity was at 83.1% (95% CI 72.2%–91.1%) and specificity at 87.2% (95% CI 74.4%–95.2%) using Youden’s index, as shown in Figure 3 (coordinates for the ROC curves are given in Supporting File 2). The Index of Union criterion also provided the same level of diagnostic accuracies as of Youden’s index. Coordinates of the ROC curves using both methods are given in Supporting File 2. An AUC of 0.922, relative to the Pelli–Robson chart, indicates that the CamBlobs charts demonstrate a comparable level of performance in measuring CS among people with glaucoma.

## 4. Discussion

The current study compares CS measurements with CamBlobs and near Pelli–Robson CS charts. Compared to age‐matched controls in participants with glaucoma, CS was significantly lower in participants with glaucoma. A strong agreement was observed between CamBlobs and Pelli–Robson charts in the assessment of CS among age‐matched controls and participants with glaucoma. The substantial overlap of the AUC estimates for the two charts provides no evidence of a statistically significant difference in their performance on measuring CS. The MD estimates obtained using 24‐2 VF protocol were also positively correlated with the CS measurements from both the charts, further supporting the comparable performance of CamBlobs chart relative to the established Pelli–Robson test.

It has been an established finding that there is a loss of CS in glaucoma, even in the early stage [24]. Previous studies have reported a more pronounced CS loss in patients with glaucoma when compared to visual acuity, which was shown by a significant correlation with the MD from VF measurements. Retinal ganglion cells (RGCs) are found to respond to contrast targets, and hence, any degeneration in the RGCs, as in glaucoma, is expected to cause reduced CS [24–26]. CS measured using the CamBlobs chart was found to be reliable compared with the near Pelli–Robson CS chart in our study. ROC modelling and analysis demonstrated that the CamBlobs chart exhibits moderate‐ to high‐level diagnostic performance in measuring CS, comparable to the Pelli–Robson chart, in both glaucomatous and nonglaucomatous eyes.

Apart from the CS differences in glaucoma and control participants which are an expected outcome, the main aim of the current study was to evaluate the utility of the CamBlobs CS compared to Pelli–Robson as the current reference standard. On evaluating the agreement between CamBlobs chart with the near Pelli–Robson chart, we found a strong agreement between the mean CS measures for the control (−0.1 log dB) and glaucoma groups (−0.06 log dB) with LOA of 0.28 log dB for the control group and 0.56 log dB for the glaucoma group (Table 2).

CamBlobs chart designs used in the current study are suitable for people with lower literacy. The chart is subject to limitations, including the fact that, as it is disposable, it may attract dust and finger smudges and therefore must be used only once. One big limitation is that we do not have many people in our current study, but this could be basis for a much larger and longitudinal study. There is a possibility of not achieving the optimum near‐vision distance in each study participant, as the CamBlobs CS testing was carried out at 40 cm, as recommended by the manufacturer.

One limitation of this study is the relatively small sample size at eye level, which consequently leads to further reduction in statistical power when analyses are considered at person level. This constrain may limit the generalisability of the findings. Among the 44 glaucomatous eyes, the distribution of VF severity was uneven, with relatively small numbers within each severity category. Consequently, the sample size within different severity strata was insufficient to permit stratified agreement analyses across levels of VF loss. This limits the ability to assess whether agreement between the tests varies according to disease severity. Nonetheless, as a pilot investigation, the study offers valuable preliminary insights into the usability and potential clinical applicability of the CamBlobs charts for assessing CS in individuals with glaucoma. The evidence generated in this study may inform designing and conducting a larger study.

## 5. Conclusions

Evidence generated in this study suggests that CamBlobs charts are comparable to standard near Pelli–Robson charts in measuring CS among the people with glaucoma. The CamBlobs charts represent a more pragmatic and reliable tool for assessing CS in people with glaucoma. Given their comparable performance to the Pelli–Robson chart and reduced reliance on examiner expertise, CamBlobs charts may offer particular advantages for use in occupational, home‐based and other nonclinical settings. The relative ease of administration of the CamBlobs chart supports its potential to enable wider and more accessible CS assessment, particularly beyond traditional clinical settings, although further validation in diverse settings is warranted.

**Nomenclature**
AUC: Area under the curve
CS: Contrast sensitivity
HVF: Humphrey visual filed
ROC: Receiver operating characteristics
OBA: Optical brightening agents
OCT: Optical coherence tomography
VF: Visual fields
VFI: Visual field index

## Author Contributions

Conceptualisation, design, protocol development and data collection: Shahina Pardhan, Rajiv Raman and Rekha Srinivasan; data analysis and interpretation: Suwin Hewage, Mapa Prabhath Piyasena and Shahina Pardhan; manuscript preparation and revisions: Mapa Prabhath Piyasena and Shahina Pardhan; final revisions of the manuscript and approval for submission: Shahina Pardhan, Rajiv Raman, Rekha Srinivasan, Mapa Prabhath Piyasena, John Kidd, and Suwin Hewage. All authors have made substantial contributions to this study.

## Funding

This study was supported by the Vision and Eye Research Institute, School of Medicine, Anglia Ruskin University, Young Street, Cambridge, United Kingdom.

## Disclosure

All authors have completed and submitted the ICMJE disclosure form.

## Ethics Statement

This study adhered to the principles of the Declaration of Helsinki.

## Consent

Written informed consent was obtained from all study participants.

## Conflicts of Interest

The authors declare no conflicts of interest.

## Supporting Information

Disposable printed CamBlobs charts for measuring contrast sensitivity in patients with glaucoma Supporting File 1. Figure 1: Bland–Altman graph with subcategorisation based on control and glaucoma groups. Supporting File 2. 2.1: Coordinates of the ROC curve relevant for Figure 2. 2.2: Coordinates of the ROC curve relevant for Figure 3.

## Supporting information


**Supporting Information** Additional supporting information can be found online in the Supporting Information section.

## Data Availability

The data generated may be obtained upon reasonable request to the corresponding author. The study protocol and the experimental details of the study were reviewed and approved by the Institutional Review Board and the Ethics Committee, Vision Research Foundation, Sankara Nethralaya, Chennai.
