# Supplementary material for: Disposable Printed CamBlobs Charts for Measuring Contrast Sensitivity in Patients With Glaucoma
Source: J Ophthalmol. 2026 Feb 27;2026:4199453. doi: 10.1155/joph/4199453 (PMC12949085; doi:10.1155/joph/4199453)
Supplement: Supplementary file 1 — Supporting Information Additional supporting information can be found online in the Supporting Information section. [file JOPH-2026-4199453-s001.docx]

**Title: Disposable printed CamBlob charts for measuring contrast sensitivity in patients with glaucoma**

**SUPPLEMENTARY FILES**

**Supplementary File 1:**

**Figure 1.** Bland-Altman graph with sub-categorisation based on control and glaucoma groups.

**Supplementary File 2:**

**2.1 Coordinates of the ROC Curve relevant for Figure 2**

**2.2 Coordinates of the ROC Curve relevant for Figure 3**

**Supplementary File 1 -**


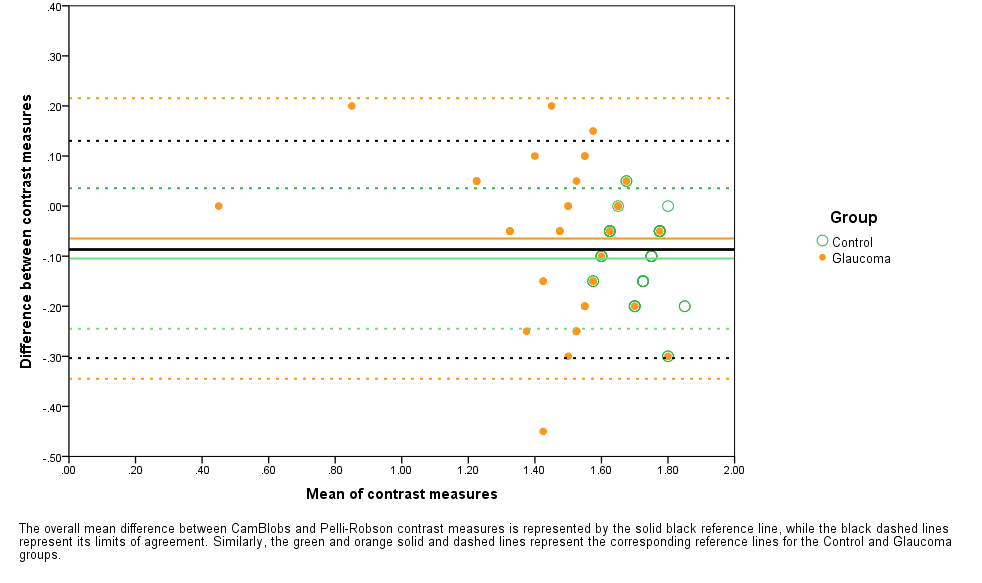


**Figure 1.** Bland-Altman graph with sub-categorisation based on control and glaucoma groups.

**Supplementary File 2 –**

**2.1 Coordinates of the ROC Curve relevant for Figure 2**

| **Area Under the Curve** | | | | | |
| --- | --- | --- | --- | --- | --- |
| Test Result Variable(s) | Area | Std. Error^a^ | Asymptotic Sig.^b^ | Asymptotic 95% Confidence Interval | |
|  |  |  |  | Lower Bound | Upper Bound |
| Reference Contrast Testing - Pelli Robson | .878 | .036 | .000 | .807 | .948 |
| Test Contrast Testing - CamBlobs | .850 | .040 | .000 | .772 | .927 |
| The test result variable(s): Reference Contrast Testing - Pelli Robson, Test Contrast Testing - CamBlobs has at least one tie between the positive actual state group and the negative actual state group. Statistics may be biased. | | | | | |
| a. Under the nonparametric assumption | | | | | |
| b. Null hypothesis: true area = 0.5 | | | | | |

**2.1.1 CamBlobs test value as index test for reference of glaucoma diagnosis based on visual field testing**

| Positive if CamBlobs value less than or equal to | Sensitivity | Specificity | Optimum cut-off decision criterion | | | |
| --- | --- | --- | --- | --- | --- | --- |
|  |  |  | **Yoden's** | Euclidean distance | **Index of Union** | Maximum product of sensitivity and specificity |
| -0.5500 | 0.0000 | 1.0000 | 0.0000 | 1 | 1.0000 | 0 |
| 0.7000 | 0.0230 | 1.0000 | 0.0230 | 0.977 | 0.9770 | 0.023 |
| 1.0750 | 0.0450 | 1.0000 | 0.0450 | 0.955 | 0.9550 | 0.045 |
| 1.2250 | 0.0680 | 1.0000 | 0.0680 | 0.932 | 0.9320 | 0.068 |
| 1.2750 | 0.1590 | 1.0000 | 0.1590 | 0.841 | 0.8410 | 0.159 |
| 1.3250 | 0.2270 | 1.0000 | 0.2270 | 0.773 | 0.7730 | 0.227 |
| 1.3750 | 0.2950 | 1.0000 | 0.2950 | 0.705 | 0.7050 | 0.295 |
| 1.4250 | 0.3640 | 1.0000 | 0.3640 | 0.636 | 0.6360 | 0.364 |
| 1.4750 | 0.5230 | 1.0000 | 0.5230 | 0.477 | 0.4770 | 0.523 |
| 1.5250 | **0.6140** | **0.9630** | **0.5770*** | 0.387769 | 0.3490 | 0.591282 |
| 1.5750 | **0.6820** | **0.8520** | 0.5340 | **0.350753** | **0.1700*** | 0.581064 |
| 1.6250 | 0.7950 | 0.6480 | 0.4430 | 0.407344 | 0.2570 | 0.51516 |
| 1.6750 | 0.9320 | 0.4260 | 0.3580 | 0.578014 | 0.5060 | 0.397032 |
| 1.7250 | 0.9770 | 0.2220 | 0.1990 | 0.77834 | 0.7550 | 0.216894 |
| 1.7750 | 1.0000 | 0.0190 | 0.0190 | 0.981 | 0.9810 | 0.019 |
| 2.8000 | 1.0000 | 0.0000 | 0.0000 | 1 | 1.0000 | 0 |

*In bold are the optimum cut-off for each decision criterion method.

**2.2.2 Pelli-Robson test value as index test for reference of glaucoma diagnosis based on visual field testing**

| Positive if Pelli-Robson value less than or equal to | Sensitivity | Specificity | Optimum cut-off decision criterion | | | |
| --- | --- | --- | --- | --- | --- | --- |
|  |  |  | **Yoden's** | Euclidean distance | **Index of Union** | Maximum product of sensitivity and specificity |
| -0.5500 | 0.0000 | 1.0000 | 0.0000 | 1.0000 | 1.0000 | 0.0000 |
| 0.6000 | 0.0230 | 1.0000 | 0.0230 | 0.9770 | 0.9770 | 0.0230 |
| 0.9750 | 0.0450 | 1.0000 | 0.0450 | 0.9550 | 0.9550 | 0.0450 |
| 1.2750 | 0.1140 | 1.0000 | 0.1140 | 0.8860 | 0.8860 | 0.1140 |
| 1.4250 | 0.2270 | 1.0000 | 0.2270 | 0.7730 | 0.7730 | 0.2270 |
| 1.5750 | 0.5000 | 1.0000 | 0.5000 | 0.5000 | 0.5000 | 0.5000 |
| **1.7250** | **0.9320** | **0.6670** | **0.5990*** | **0.3399** | **0.2650*** | **0.6216** |
| 1.8750 | 0.9770 | 0.0740 | 0.0510 | 0.9263 | 0.9030 | 0.0723 |
| 2.9500 | 1.0000 | 0.0000 | 0.0000 | 1.0000 | 1.0000 | 0.0000 |

*In bold are the optimum cut-off for each decision criterion method.

Both Yoden’s and Index of Union suggests 1.7250 as the optimum accuracy level of 0.9320 sensitivity and 0.6670 specificity for Pelli-Robson charts.

95% CI for sensitivity of 0.9320 = 0.8326 to 0.9826

95% CI for specificity of 0.6670 = 0.5353 to 0.7826

**2.2 Coordinates of the ROC Curve relevant for Figure 3 -**

| **Area Under the Curve** | | | | |
| --- | --- | --- | --- | --- |
| Test Result Variable(s): Test Contrast Testing - CamBlobs | | | | |
| Area | Std. Error^a^ | Asymptotic Sig.^b^ | Asymptotic 95% Confidence Interval | |
|  |  |  | Lower Bound | Upper Bound |
| .922 | .025 | .000 | .872 | .972 |
| The test result variable(s): Test Contrast Testing - CamBlobs has at least one tie between the positive actual state group and the negative actual state group. Statistics may be biased. | | | | |
| a. Under the nonparametric assumption | | | | |
| b. Null hypothesis: true area = 0.5 | | | | |

**CamBlobs test value as index test for reference of glaucoma diagnosis based on Pelli-Robson contrast testing cut-off value at CS Log=1.7250**

| **Positive if CamBlobs value less than or equal to** | Sensitivity | Specificity | Optimum cut-off decision criterion | | | |
| --- | --- | --- | --- | --- | --- | --- |
|  |  |  | **Yoden's** | Euclidean distance | **Index of Union** | Maximum product of sensitivity and specificity |
| -0.5500 | 0.0000 | 1.0000 | 0.0000 | 1.0000 | 1.0000 | 0.0000 |
| 0.7000 | 0.0170 | 1.0000 | 0.0170 | 0.9830 | 0.9830 | 0.0170 |
| 1.0750 | 0.0340 | 1.0000 | 0.0340 | 0.9660 | 0.9660 | 0.0340 |
| 1.2250 | 0.0510 | 1.0000 | 0.0510 | 0.9490 | 0.9490 | 0.0510 |
| 1.2750 | 0.1190 | 1.0000 | 0.1190 | 0.8810 | 0.8810 | 0.1190 |
| 1.3250 | 0.1690 | 1.0000 | 0.1690 | 0.8310 | 0.8310 | 0.1690 |
| 1.3750 | 0.2200 | 1.0000 | 0.2200 | 0.7800 | 0.7800 | 0.2200 |
| 1.4250 | 0.2710 | 1.0000 | 0.2710 | 0.7290 | 0.7290 | 0.2710 |
| 1.4750 | 0.3900 | 1.0000 | 0.3900 | 0.6100 | 0.6100 | 0.3900 |
| 1.5250 | 0.4920 | 1.0000 | 0.4920 | 0.5080 | 0.5080 | 0.4920 |
| 1.5750 | 0.6440 | 1.0000 | 0.6440 | 0.3560 | 0.3560 | 0.6440 |
| **1.6250** | **0.8310** | **0.8720** | **0.7030*** | **0.2120** | **0.0410*** | 0.7246 |
| 1.6750 | 0.9320 | 0.5640 | 0.4960 | 0.4413 | 0.3680 | 0.5256 |
| 1.7250 | 1.0000 | 0.3330 | 0.3330 | 0.6670 | 0.6670 | 0.3330 |
| 1.7750 | 1.0000 | 0.0260 | 0.0260 | 0.9740 | 0.9740 | 0.0260 |
| 2.8000 | 1.0000 | 0.0000 | 0.0000 | 1.0000 | 1.0000 | 0.0000 |

*In bold are the optimum cut-off for each decision criterion method.

All methods suggest 1.6250 as the optimum cut-off for Camblobs, with 0.8310 sensitivity and 0.8720 specificity
